# A Rare Case of Neonatal Cholestasis Linked to FOCAD Gene Variants: Exploring the Variable Phenotypic Presentation and Its Implications

**DOI:** 10.1155/crig/9569160

**Published:** 2025-07-01

**Authors:** Ariel Tarrell, Jessika Weber, Reem Shawar, Luca Brunelli, Susan Morelli, Pinar Bayrak-Toydemir, Elizabeth Doughty, Gulsen Akay, Lorenzo D. Botto, Emily Flemming, N. Scott Reading, Catalina Jaramillo

**Affiliations:** ^1^Department of Pediatrics, Division of Neonatology, Primary Children's Hospital, University of Utah School of Medicine, Salt Lake City, Utah, USA; ^2^Department of Pediatrics, Division of Pediatric Endocrinology, Primary Children's Hospital, University of Utah School of Medicine, Salt Lake City, Utah, USA; ^3^Intermountain Health, Salt Lake City, Utah, USA; ^4^Department of Pathology, University of Utah School of Medicine, Salt Lake City, Utah, USA; ^5^Department of Pediatrics, Division of Medical Genetics, University of Utah School of Medicine, Salt Lake City, Utah, USA; ^6^Department of Pediatrics, Division of Neurology, University of Utah School of Medicine, Salt Lake City, Utah, USA; ^7^Department of Internal Medicine, Division of Hematology, University of Utah School of Medicine, Salt Lake City, Utah, USA; ^8^ARUP Laboratories, Salt Lake City, Utah, USA; ^9^Department of Pediatrics, Division of Pediatric Gastroenterology, Hepatology and Nutrition, Primary Children's Hospital, University of Utah School of Medicine, Salt Lake City, Utah, USA

## Abstract

Neonatal liver disease is a broad entity. When it presents in conjunction with other abnormalities, it raises the question of a potential underlying genetic cause. Etiologies that were once difficult to diagnose are becoming more readily identifiable with the arrival of next-generation sequencing. We present a rare cause of neonatal liver disease, a FOCAD gene variant, that was determined to be the most likely cause of an infant's liver disease and other findings. This case adds to only a few reports in the literature on this presentation in the neonatal period.

## 1. Introduction

Neonatal liver disease encompasses a wide spectrum of diagnostic considerations including mild physiologic conditions, infections, metabolic conditions, ischemia, and fulminant liver failure [1]. Fortunately, advances in genetic sequencing have greatly improved the ability to diagnose, treat, and prognosticate rare or difficult-to-diagnose conditions [2–4]. We present a case of an infant with a constellation of findings, including cholestatic hepatopathy and hypoglycemia, attributed to a suspected notable genetic variant identified with the use of next-generation sequencing (NGS).

## 2. Case Report

A growth-restricted Hispanic male infant was delivered prematurely via cesarean section at 36 weeks of gestation for decreased fetal heart tones. He was born small for gestational age (birth weight *Z*-score: -3.34). His mother had pyelonephritis early during pregnancy but no other complications. She did have a history of a prior premature birth and an abortion. Maternal and paternal family history was unrevealing, and the patient had a healthy older sister. He presented to the neonatal intensive care unit with hypoglycemia requiring several dextrose boluses and intravenous fluids. The exam included a large anterior fontanelle, mildly low-set ears, a long philtrum, and a right inguinal hernia.

### 2.1. Liver/Gastroenterology Findings

**Figure 1:**
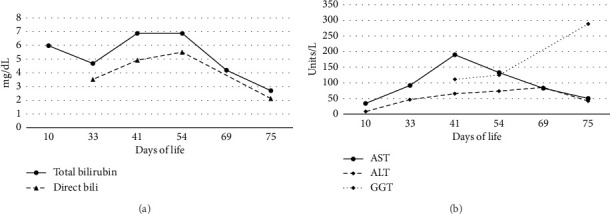
Laboratory values: (a) total and direct bilirubin trends, (b) alanine aminotransferase, aspartate aminotransferase, and gamma-glutamyl transferase values.

**Figure 2:**
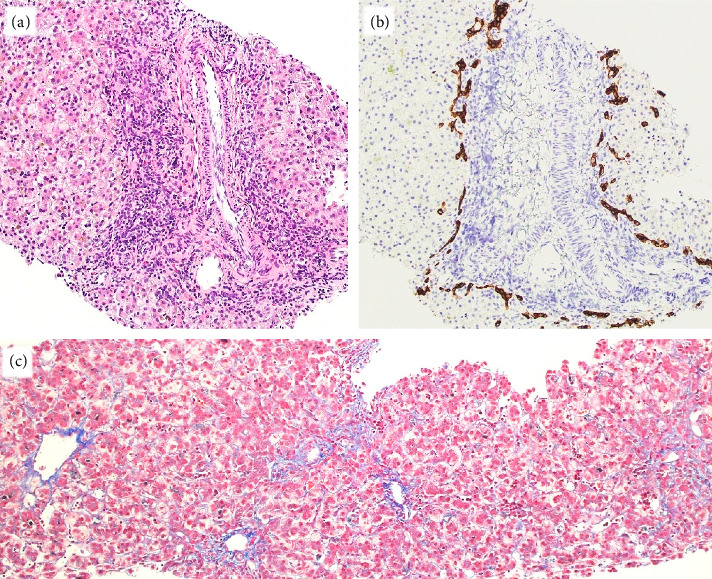
Liver biopsy: (a) H&E, 100x–portal triad with mild mixed inflammatory infiltrate in a background of cholestatic hepatopathy (b) CK7, 100x–portal triad with peripheral bile ductular proliferation and central bile duct paucity highlighted by immunostain (c) trichrome, and 100x–mild focal and periportal fibrosis.

On Day-of-life (DOL) 5, the total bilirubin level peaked at 13.6 mg/dL, which improved with phototherapy. Due to ongoing hyperbilirubinemia, further evaluation revealed elevated direct bilirubin of 5.3 mg/dL on DOL 9. Liver enzyme elevation with aspartate aminotransferase 190 μ/L, alanine aminotransferase 66 μ/L, and gamma-glutamyl transferase of 135 μ/L was noted (Figure 1). Liver function was normal with an international normalized ratio of 1.0. New-onset clay-colored stool on DOL 35 prompted a liver ultrasound which showed a contracted, thick-walled gallbladder and normal liver parenchyma appearance. A percutaneous transhepatic cholangiogram showed a diminutive intrahepatic biliary system with drainage of the extrahepatic biliary system to the duodenum. Liver biopsy results are shown in Figure 2. Liver biopsy showed a cholestatic pattern of injury in a background of central bile duct paucity and patchy triaditis. A reactive bile ductular proliferation and mild fibrotic expansion involved the periportal space in a patchy distribution. In addition to the absence of traditional features of biliary obstruction, such as bile duct plugging, no significant lobular inflammation, necrosis, giant cell change, or steatosis were identified. Eventually, the acholic stool resolved. Pertinent results included negative cytomegalovirus testing, a normal ammonia level, satisfactory TSH and free T4 levels, normal alpha-1 antitrypsin level and phenotype, and negative maternal prenatal labs. There was no TPN exposure, no feeding intolerance, and no family history of liver disease.

### 2.2. Endocrinology Findings

The course was notable for hypoglycemia at birth which persisted beyond DOL 3. Hypoglycemia evaluation confirmed a low serum glucose with inappropriately elevated insulin, suppressed beta-hydroxybutyrate, and a positive glycemic response to glucagon consistent with hyperinsulinism. Diazoxide and thiazide were started but he continued to have hypoglycemia and developed fluid retention. Euglycemia was achieved with parenteral and eventually, dextrose solution via nasogastric tube. A gastrostomy tube was placed for nutritional support to maintain euglycemia. An 18F-DOPA PET scan demonstrated an overall diffuse look with the pancreas appearing slightly prominent and bulky. Genetic causes of hyperinsulinism were ruled out.

### 2.3. Genetic Findings

Other testing included normal microarray, methylation studies for Beckwith–Wiedemann and Russell–Silver syndrome, and state newborn screening. A “critically ill rapid genetic diagnostic panel” was obtained on the infant and both parents due to the lack of a unifying diagnosis. We used a human, nuclear, inherited disease panel with biotinylated DNA probes designed to target 4503 disease-causing OMIM database genes. A single capture was used to create the proband's and parent's genomic library. After sequencing, the FASTQ files were processed with an internal bioinformatics pipeline and proprietary software was used for alignment and variant calls. The probands' variants were analyzed to search for any variants potentially associated with reported clinical phenotypes using HPO terms. All variants were also analyzed under inheritance patterns. The panel showed compound heterozygous variants of uncertain significance (VUS) in the FOCAD gene and a compound heterozygous pathogenic variant and VUS in the DOCK6 gene (Table 1). As findings of Adams–Oliver syndrome-2, such as limb or skin anomalies, were not present in the patient, the DOCK6 variants were deemed not to contribute to the patient's phenotype. Targeted sequencing on the patient's phenotypically healthy sibling did not show either FOCAD variant. The c.1532A > G; p.Tyr511Cys variant has been reported only 6 times in a population database [5]. There is a large physicochemical difference between Tyr and Cys. The other variant, c.5137C > A; p.Pro1713Thr, has been reported only once in population databases; however, conservation and physicochemical differences are not as profound as the Tyr511Cys variant [5]. Since both variants have never been reported previously on affected individuals, the classification of the variants currently remains as VUS based on guidelines [6].

At around 4 months of age, the patient underwent a safety fast, in which he was off oral/enteral feeds and off dextrose-containing fluids for 8 h. He remained euglycemic consistent with transient neonatal hyperinsulinism that resolved. By 6 months, the patient had a resolution of cholestasis and no worsening liver disease. The growth chart was notable for overall slow growth; weight remained small for gestational age, though it did increase slowly toward the fifth percentile by 5 months of age. The gastrostomy tube was removed at 8 months of age. The patient will follow up with multiple subspecialists for surveillance of other features of the FOCAD variant.

## 3. Discussion

We describe a rare case of FOCAD variants associated with liver disease in a premature infant without liver failure or cirrhosis. FOCAD encodes focadhesin, a poorly described protein whose structure and function are not well established. However, recent genetic and clinical data support that loss-of-function mutations in FOCAD can lead to Mendelian syndrome in children. Furthermore, animal models and *in vitro* functional tests using patient-derived and disease-relevant human cell lines have established a link between FOCAD deficiency and neonatal liver cirrhosis. Specifically, it has been found that the absence of FOCAD impacts the SKIC2 and SKIC3 protein levels. These proteins are two essential components of the SKI mRNA surveillance pathway, which is an important genetic network to preserve liver homeostasis [7].

FOCAD variants have been reported in cases of pediatric cirrhosis and liver failure, and although there are limited data regarding this genetic variant, an international case series described 14 cases with variable hepatic and extrahepatic abnormalities. Of the cases, 100% of individuals had a hepatic abnormality, and it was the organ that was the most severely affected. Cirrhosis, hepatomegaly, hypoalbuminemia, and elevated liver enzymes were present in 71.4%. Intrahepatic cholestasis was identified in 35.7%. Portal hypertension complications were also noted with splenomegaly detected in 71.4% and ascites in 28.6%. Only one patient underwent liver transplantation at 6 months of age but subsequently died as a result of multiorgan dysfunction. Furthermore, intrauterine growth failure (71%), inguinal hernia (42%), feeding difficulties (42%), and hyperinsulinemic hypoglycemia (36%) were also reported, which are found in this patient's presentation. Six of the 14 cases (43%) died during infancy or childhood. Five of these children were under the age of 1 year [7].

This patient's milder phenotype may be explained by an earlier identification in a young infant; thus, it is possible that the liver disease may progress. Alternatively, the patient may have an inherently milder form of FOCAD-associated disease, whose phenotypic spectrum is not fully known. It is likely that the neonatal liver disease was related to the FOCAD variant, though ultimately this cannot yet be definitively proven. The variants were reported as VUS, indicating that convincing evidence is still lacking as to whether they are disease-causing (pathogenic, or likely pathogenic) or not (benign, or likely benign) [6]. However, the balance of the evidence, including the extensive workup, the absence of other explanations, and the findings that aligned with other described cases of FOCAD variants, suggests that this patient has a relatively mild form of FOCAD-associated disease, thus expanding the phenotypic spectrum.

This case highlights how rapid NGS in diagnostic uncertainty is the key to the evaluation of infants with unexplained liver disease. This diagnosis of an apparently ultrarare condition would have been exceedingly challenging without the availability of NGS. Awareness of FOCAD, its variable phenotypic presentation, and hepatic involvement is warranted. Even with a milder phenotype in this patient, close follow-up and consideration for progression of liver disease are needed because of the potential risk of evolving fibrosis and clinically significant liver disease. In conclusion, FOCAD variants should be a diagnostic consideration in patients undergoing NGS for liver disease and multiorgan system involvement. Further investigation of the significance of FOCAD variants and related pathology is warranted.

## Figures and Tables

**Table 1:** Results of the whole-exome sequencing: two genes with variants were identified, with the FOCAD variant being a variant of unknown significance.

Gene (transcript)	Condition	Inheritance	Variant	Genomic coordinates	Classification	Frequency gnomAD MAF	Revel
FOCAD (NM_017794.5)	Liver disease, severe congenital (MIM: 619991)	Autosomal recessive	c.1532A > G; p. Tyr511Cys (paternal)	Chr (9) (GRCh37): g.20819871	VUS	0.0025%	0.204
FOCAD (NM_017794.5)	Liver disease, severe congenital (MIM: 619991)	Autosomal recessive	c.5137C > A; p.Pro1713Thr (maternal)	Chr (9) (GRCh37): g.20990254	VUS	0.0012%	0.011
DOCK6 (NM_020812.4)	Adams–Oliver syndrome-2 (MIM: 614219)	Autosomal recessive	c.5939 + 2T > C (maternal)	Chr (19) (GRCh37): g.11311390	Pathogenic	0.021%	N/A
DOCK6 (NM_020812.4)	Adams–Oliver syndrome-2 (MIM: 614219)	Autosomal recessive	c.1963G > A; p.Gly655Ser (paternal)	Chr (19) (GRCh37): g.11348325	VUS	Not in gnomAD	0.608

## Data Availability

The data that support the findings of this study are available on request from the corresponding author. The data are not publicly available due to privacy or ethical restrictions.
